# A simple tool to evaluate common disorders: validation of a “proctological symptom scale”

**DOI:** 10.1007/s00384-015-2160-7

**Published:** 2015-02-20

**Authors:** Matthias Kraemer, David Kara, Michael Rzepisko, Joel Sayfan

**Affiliations:** 1 Department of General and Visceral Surgery, Coloproctology, St. Barbara-Klinik Hamm, Germany; 2 Department of Trauma and Orthopedic Surgery, St. Barbara-Klinik Hamm, Germany; 3 Colorectal Surgery, Hertzeliya Medical Center (HMC), Hertzeliya, Israel

**Keywords:** Proctology, Visual scale, Scores, Haemorrhoidal disease

## Abstract

**Purpose:**

Proctological symptomatology is of little complexity and therefore appears particularly suitable for comparative evaluation by visual scales. We devised a “proctological symptom scale” (PSS) with separate scales for four cardinal proctological symptoms: pain, itching/irritation, discharge/moisture, and bleeding. The objective of this study was to evaluate the PSS among proctological patients and non-proctological controls.

**Methods:**

This was a single center non-interventional observational study on 229 proctological patients and 133 controls. The main outcome measures investigated were age- and sex-stratified comparison of the non-proctological cohort and the controls, effect of therapeutic intervention on scale values in a subset of patients with haemorrhoidal disease, and sensitivity of the PSS to detect therapeutic failure in this subset of patients.

**Results:**

The PSS was found to significantly differentiate between proctological patients and controls. Gender and age had no significant influence on PSS values in the proctological cohort. The intervention (one session of rubber band ligation in patients with haemorrhoidal disease) was reflected by a significantly improved overall PSS. In 16 cases within this group, the PSS got worse. A case-by-case follow-up of these patients showed that 14 of the 16 patients ended up with surgery (or with the advice to have surgery).

**Conclusions:**

The PSS reliably differentiates proctological patients from non-proctological controls. Following intervention, the PSS reliably differentiated therapeutic success from failure. We find the PSS to be a simple and useful tool in our clinical routine since it provides an easily obtainable and reproducible basis for the visit-by-visit assessment of proctological patients. The PSS may also be suitable for studies to measure and compare symptomatic improvement and success of different therapies in proctology.

## Introduction

Proctological complaints are common. Treatment modalities range from self-treatment with various ointments and suppositories to specialised procto-surgical interventions. Although no reliable data are available, unquestionable proctological disorders have a sizeable economic impact. Hence, a simple tool to identify useful and eliminate less useful therapeutic options seems desirable.

Proctological symptomatology is of little complexity and therefore appears particularly suitable for simple comparative assessment. The idea of a symptom scale was first devised by Sayfan et al. in studies focussing on the outcome of operative therapy of haemorrhoidal disease [1]. The authors used visual analogue scales (VAS) to measure the severity of five major anal symptoms (pain, itching, discharge/moisture, bleeding, prolapse). They were able to demonstrate a significant impact of the intervention on results. However, the instrument was never tested on controls and the idea of expanding its use is not pursued further.

## Materials and methods

Following a pilot phase of haphazard sampling of the original symptom scale [1] among a series of 205 patients presenting to our proctological outpatient clinic, we found that “prolapse” as scale item was almost never chosen (<5 %). Although we initially found this to be quite perplexing, we eventually decided to exclude this item to keep the scale as simple as possible. The reason for the lack of impact of this item may be that “prolapse” is a symptom pertaining exclusively to a small fraction within the group of patients with haemorrhoidal disease. On the other hand, the “proctological symptom scale” (PSS) was used on the entire spectrum of benign proctological disorders.

**Fig. 1:**
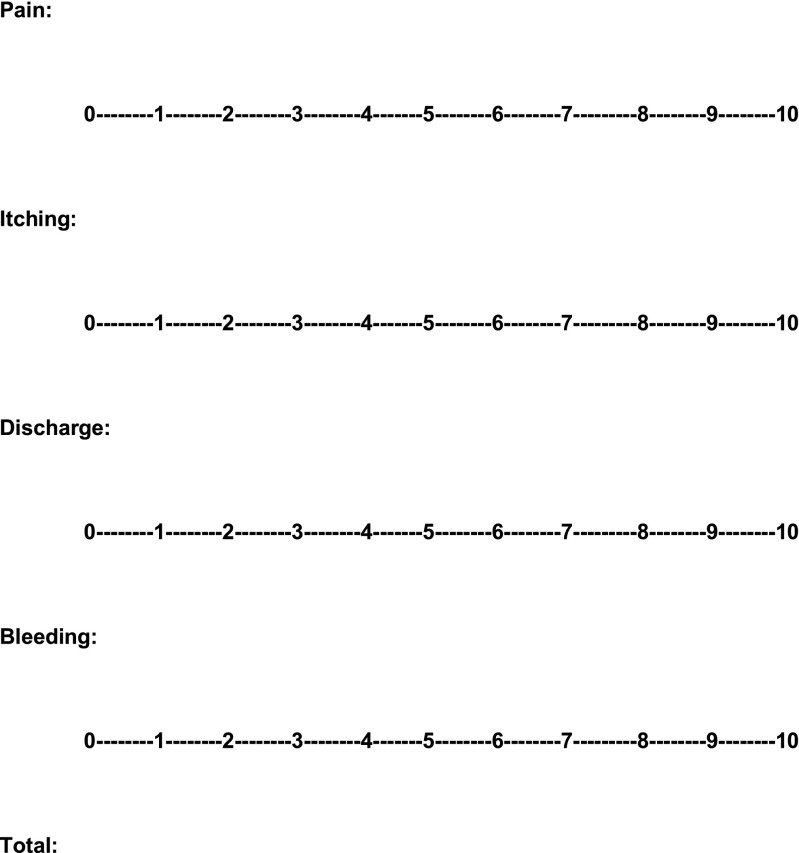
The proctological symptom scale

The PSS therefore comprises four items: anal pain, anal itching/irritation, anal discharge/moisture, anal bleeding. Patients are asked to mark the severity they subjectively attribute to their symptom anywhere along the line on a 0 to 10 scale for each item separately (Fig. 1). To facilitate the use of the scale for both patient and clinician, we decided to mark the intervals from 0 to 10 and not use the classic visual analogue scale design without such markings. This scale enables the clinician to calculate the scale value without the use of a ruler. Scale markings in between whole numbers were rounded up or down to the nearest whole number. Markings halfway in between the numbers were rounded up.

To establish the age- and sex-dependent variance among non-proctological individuals, the PSS was first tested on unselected hospital staff and patients, who attended our surgical outpatients department for complaints such as hernias or cholecystitis. One series was taken among patients of a specialised traumatological-orthopedic department.

To test the PSS in a proctological setting, we chose unselected patients presenting to our proctological outpatient clinic. Patients were also asked to fill in the standard constipation [2] and incontinence [3] scores in addition to the PSS. We then focused on the more defined subgroup of patients with symptomatic haemorrhoidal disease suitable for Barron ligation within this cohort. At first presentation, the diagnosis was established and Barron ligations were explained and suggested as therapy. Patients were then supplied with a consent form and a re-appointment was made for the first treatment. Patients were asked to fill in the PSS at first presentation and every ensuing visit. The time interval between the Barron ligations usually is 4 to 6 weeks.

The sampled PSS data were analysed as follows:

- Age- and sex-stratified comparison of scale values of the non-proctological cohort and the proctological cohort at first presentation. The relative impact of each scale item for the overall results was analysed separately by comparison of the positive response rates (i.e. item marked >0) for each item.
- Effect of intervention (Barron ligation) on scale values in the subset of patients with haemorrhoidal disease. This was done by comparative analysis of the PSS baseline values (prior to first ligation) with the PSS at the time of presentation for the second ligation.
- Within the same subset of patients with haemorrhoidal disease the sensitivity of the PSS to detect therapeutic failure was done by an additional chart review of cases whose overall scale value got worse following their first Barron ligation. Their eventual outcome was assessed following all further ligations.
- To find out whether the PSS could in part or entirely be replaced by the widely used constipation [2] or incontinence scores [3], the three instruments were compared in 100 unselected and consecutive proctological patients. If patients responded “0” in either of the scores, the score was considered as “blind” to the patient’s symptomatology. Patients scoring “0” in either of the scores were therefore dichotomized and the respective PSS response in this group was analysed.

Statistical analyses were done by *T* test calculation using SPSS keg1 statistical software (SPSS Inc., Chicago, IL). Statistical significance was achieved with *P* < 0.05.

Approval for this study was obtained by the responsible ethic board.

## Results

In all, 229 proctological patients were compared to 133 controls. The PSS was found to significantly differentiate between both groups (Table 1). Whereas in the control group, the PSS showed significantly higher values in males; no gender difference was found in the proctological cohort (Table 2). Also, PSS values of younger and of older proctological patients did not vary significantly (Table 3). Positive scale values (i.e. scale values above “0”) were recorded in 86 % of the patients for “itching”, 75 % for “discharge”, 68 % for “pain”, and 52 % for “bleeding”, reflecting the respective weight of each item for the PSS. One session of rubber band ligation in the subgroup of patients with haemorrhoidal disease was reflected by a significantly improved overall PSS in this group (Table 4). However, 16 cases within this group registered with a worse PSS. A case-by-case follow-up of these patients (Table 5) showed that 14 of the 16 patients eventually ended up with surgery (or with the advice to have surgery). Among the 100 patients who in addition to the PSS had the constipation and incontinence scores analysed 12 % scored “0” in the constipation score, 46 % scored “0” in the incontinence score. Patients marking “0” in their constipation score had an average PSS of 6 (median 5, range 0–16), patients marking “0” in their incontinence scores had an average PSS of 10 points (median 7, range 0–28).

**Table 1:** Scale point distribution in proctological patients and in controls

	Proctological patients (*n* = 229)	Controls (*n* = 133)	*P* value
Scale points^a^ mean/median (range)	10/9(3–40)	2/1(0–14)	<0.001

^a^Maximum scale points: 40

**Table 2:** Scale point distribution vs. gender in proctological patients and in controls

Proctological patients			*P* value
Gender	Male	Female	
	(*n* = 147)	(*n* = 84)	
Scale points^a^ mean/median (range)	11/10(3–40)	9/8(3–30)	0.111
Controls			
Gender	Male	Female	
	(*n* = 73)	(*n* = 60)	
Scale points^a^ mean/median (range)	3/2(0–14)	2/2(0–7)	0.01

^a^Maximum scale points: 40

**Table 3:** Scale point distribution vs. age in proctological patients

Proctological patients			*P* value
Age (years)	≤50	50+	
	(*n* = 114)	(*n* = 115)	
Scale points^a^ mean/median (range)	11/10(3–30)	11/10(3–40)	0.755

^a^Maximum scale points: 40

**Table 4:** Overall impact of one application of rubber band ligation on scale values in a cohort of patients with hemorrhoidal disease (*n* = 104)

	Baseline	First ligation	*P* value
Scale points^a^ mean/median (range)	10/9(3–25)	7/6(0–25)	<0.001

^a^Maximum scale points: 40

**Table 5:** Case-by-case analysis of patients registering worse scale values following first application of rubber band ligation (*n* = 16)

	Baseline	First ligation	Eventual outcome
M, 31	19	22	Operation
F, 55	6	9	Operation
F, 62	9	15	Operation
M, 61	20	21	Operation
F, 52	10	14	Operation
F, 54	5	7	Operation
F, 71	11	15	Operation
M, 46	5	12	Operation
M, 46	8	9	Operation advised
M, 58	12	15	Operation advised
F, 60	8	15	Operation advised
M, 82	8	10	Operation advised
M, 69	3	5	Operation advised
M, 35	8	10	Operation advised
M, 38	23	33	Improved with further ligations
F, 58	3	9	Improved with further ligations

## Discussion

The proctological symptom scale is a simply structured tool for the assessment of proctological complaints. It measures the four cardinal proctological symptoms by visual analogue scales for each symptom separately. The scale can be used for every patient visit. Patients need a few seconds to fill it in; doctors require a few seconds to calculate the scale value. The scale gives an objective measure of the severity of complaints and of our therapeutic performance. In clinical routine, the PSS clearly offers a better and more detailed basis for the evaluation of symptom progress than routine entries found in most outpatient charts (such as “improved” or “feels better”).

The proctological symptom scale is the first instrument to focus exclusively on anal complaints. It was devised as a concomitant tool for daily proctological routine. Scores commonly used in the coloproctological setting are considerably more intricate and aim at more complex complaints such as incontinence and constipation [2–6]. They may incorporate quality of life issues [3, 4]. The PSS does not replace the established scores neither is the PSS replaced by the scores.

The PSS reliably differentiates proctological patients from non-proctological controls. Following intervention, the PSS reliably differentiated therapeutic success from failure. This proved significant in our subanalysis of “early failures” following rubber band ligation. The further course eventually led to therapeutic changes in most cases in this subgroup of patients. In this context, it is notable that these “failures” were detected very early in the course of their treatment (after their first rubber band ligation), which may imply a certain prognostic value of the PSS.

We find the PSS to be a simple and useful tool in our clinical routine since it provides an easily obtainable and reproducible basis for the visit-by-visit assessment of proctological patients. The PSS may also be suitable for studies to measure and compare symptomatic improvement and success of different therapies in proctology. This may help to disentangle the plethora of options offered by the industry.
